# Sol-gel Synthesis, Photo- and Electrocatalytic Properties of Mesoporous TiO_2_ Modified with Transition Metal Ions

**DOI:** 10.1186/s11671-017-2002-3

**Published:** 2017-03-31

**Authors:** N. Smirnova, I. Petrik, V. Vorobets, G. Kolbasov, A. Eremenko

**Affiliations:** 1 O.Chuiko Institute of Surface Chemistry, Ukrainian National Academy of Sciences, 17 General Naumov Street, Kyiv, 03680 Ukraine; 2 Institute of General & Inorganic Chemistry of National Academy of Sciences of Ukraine 32/34 Acad, Palladin Street, Kyiv, 03680 Ukraine

**Keywords:** Mesoporous TiO_2_, Nanosized films, Doping with transition metal ions, Cr^VI^ photoreduction, Electrocatalytic oxygen reduction, 82.45.Mp, 82.45.-h, 82.50.Hp

## Abstract

Mesoporous nanosized titania films modified with Co^2+^, Ni^2+^, Mn^3+^, and Cu^2+^ ions have been produced by templated sol-gel method and characterized by optical spectroscopy, X-ray diffraction (XRD), and Brunauer, Emmett, and Teller (BET) surface area measurement. Band gap energy and the position of flat band potentials were estimated by photoelectrochemical measurements. The films doped with transition metals possessed higher photocurrent quantum yield, as well as photo- and electrochemical activity compared to undoped samples. M^n+^/TiO_2_ (M–Co, Ni, Mn, Cu) electrodes with low dopant content demonstrate high efficiency in electrocatalytic reduction of dissolved oxygen. Polarization curves of TiO_2_, TiO_2_/Ni^2+^, TiO_2_/Co^2+/3+^, and TiO_2_/Mn^3+^ electrodes contain only one current wave (oxygen reduction current). It means that reaction proceeds without the formation of an intermediate product H_2_O_2_.

## Background

Among the semiconductor materials, titanium dioxide attracts the great attention because of its chemical stability, biological inertness, low toxicity, and relatively low cost. TiO_2_ is a promising material for application in environmental photocatalysis, for the generation of electricity in the solar and fuel cells, gas sensors, optical and protective coatings, electrochemical devices, etc. [1, 2]. Nowadays, the efforts of researchers are directed on the selection of modifiers that would expand the functionality of TiO_2_ coatings without reducing their photocatalytic activity. For example, transition metals such as Co, Ni, and Mn are well known as magnetic materials, copper and many of its compounds have antibacterial properties. As was reported by D. Banneman [3], modification of TiO_2_ nanoparticles with d-metals having an unpaired electron leads to increase the photocatalytic activity. Doping with Co, Ni, and Mn ions increases the photocatalytic activity of TiO_2_ powders and changes magnetic properties of such materials [4]. The copper ion’s ability to increase the photocatalytic activity of titanium dioxide under UV irradiation in reaction of metallic copper deposition, decomposition of formic acid, cyanide and cyanate [5, 6] connected with the efficient capturing the electrons of the TiO_2_ conduction band on Cu^2+^ and Cu^+^ energy levels near conductive band in the band gap of TiO_2_. The increase of catalytic activity in photooxidation reactions can occur in the presence of Cu^2+^ ions through more efficient formation of H_2_O_2_ and active OH^*^ radicals [7]. At the same time, decrease of the photoactivity of M^n+^–TiO_2_ materials prepared by ion beam-induced CVD in comparison with TiO_2_ was reported in [8].

Compared with industrial production [6], the sol-gel synthesis is a simple, relatively inexpensive, and reliable method for receiving of mesoporous materials [9, 10] that achieves high chemical homogeneity of formed products with significant decrease of the heating temperature and duration of heat treatment. This is a promising approach for the synthesis of powders and films of individual oxides [11] and mixed oxide materials [12, 13], production of nanostructured TiO_2_ doped with nanoparticles of noble metals [14], or transition metal oxides, due to a number of its advantages: the possibility of controlling the size of the crystals and the phase composition, the production of transition crystal structures, and the formation of high surface area.

In this paper, template sol-gel synthesis of mesoporous titanium dioxide films modified with transition metal ions (cobalt, nickel, manganese, and copper) is reported. Phase composition, optical properties, and energy parameters (band gap E_g_ and flat band potential E_fb_) of the resulting materials are investigated. The aim of this work is to study the effect of 3d-metal dopants in TiO_2_ films on their photo- and electrocatalytic properties on the example of the photoreduction reaction of potassium dichromate under UV irradiation and electroreduction of oxygen, which is the basis of electrochemical sensors of O_2_, that today are widely used to determine the concentration of dissolved oxygen as in industrial processes and in medical practice.

## Methods

Mesoporous TiO_2_, Co^n+^/TiO_2_, Ni^n+^/TiO_2_, Mn^n+^/TiO_2_, and Cu^n+^/TiO_2_ films and powders were synthesized via templated sol-gel method according to [14] using Ti(OiPr)_4_, CuSO_4_ · 5H_2_O, Co(CH_3_COO)_2_ · 4H_2_O, Ni(HCOO)_2_ · 2H_2_O, MnCl_2_ · 4H_2_O as titania and 3d-metal sources, Pluronic P123 as template, and acetylacetone as complexing agent. The molar ratios of the components were as follows: Pluronic P123:acetylacetone:HNO_3_:Ti(OiPr)_4_ = 0.1:1:2:2. For film deposition onto glass or titanum substrates, dip-coating technique was utilized. After deposition of the film, gelation, and gel ripening, it was dried in air at room temperature for 2 h. The dried films were sintered at 400 °C. At this temperature, the ordered porous structure of the oxide film is formed. To facilitate structural investigations by X-ray diffraction (XRD), corresponding powders have been prepared via gelation of the films’ precursors, their drying in air with following heat treatment at 450 or 650 °C. XRD measurements were performed using a DRON-4-07 (Burevestnik, St. Petersburg) diffractometer (CuKα radiation with Ni filter) with Brag-Brentano registration geometry (2*θ* = 10–60^o^). The average size of crystallites was determined using Scherrer equation applied to the most intensive peak. The degree of the powder crystallinity was estimated as the ratio of integrated intensities, such as for the (101) line of the studied and reference standard samples (reference standard: TiO_2_, anatase 100%). Optical spectra of the films and powders were recorded using a Perkin-Elmer Lambda Bio 35 UV-Vis with integrating sphere Labsphere RSA-PR-20 in spectral diapason 200–1000 hm. The film thickness and refractive index were measured using a multi-angle ellipsometer LEF-3M (λ = 632.8 nm).

Photoelectrochemical investigations of the TiO_2_ and M^n+^TiO_2_ electrodes were carried out in the wavelength range 250–600 nm in a quartz electrochemical cell under irradiation of a high-pressure xenon lamp, which gave light with a frequency of 20 Hz passing through a monochromator with spectral resolution of 1 nm and focused on the semiconductor electrode. The *i*_ph_ spectra were expressed in units of quantum efficiency (electron/photon). Ag/AgCl electrode was used as the reference electrode on the pH value of the electrolyte. Electrocatalytic activity of M^n+^/TiO_2_ films in the process of oxygen reduction was studied by means of the current-voltage dependencies measured in potentiodynamic mode using a specially designed electrochemical stand with the following characteristics: measured currents 2 × 10^−9^ ÷ 10^−1^ A, speed of the potential sweep 0.01 ÷ 50 mV/s, and the working range −4 ÷ +4 V.

Photocatalytic activity of the synthesized films has been checked in the process of Cr(VI) to Cr(III) photoreduction in water solution of K_2_Cr_2_O_7_ (*C*_M_ *=* 2 × 10^*−*4^ M) in the presence of electron donor EDTA (*C*_M_ *=* 2 × 10^*−*4^ M) at pH *=* 2. The open reactor with the reaction components (enabled continues inflow of oxygen) was irradiated with an UV light of mercury lamp PRK-1000 with *P*_0_ *=* 3 × 10^*−*7^ einstein dm^*−*3^ s^*−*1^ intensity. Running water was circulated through the jacket to ensure constant temperature of the magnetically stirred reaction mixture. During the experiments, concentration of reagents has been controlled with an UV-VIS spectrometer Perkin-Elmer Lambda-35.

## Results and Discussion

### Crystalline Structure of M^n+^/TiO_2_ Nanocomposites

The diffraction reflex at low 2*θ* = 2 values in the diffraction patterns of the initial films and powders correspond to the ordered mesostructures formed by the template Pluronic. The absence of this reflex in the diffraction patterns of calcined powders indicates the disordering of organized structure in the process of crystallization of titanium dioxide. Investigation of the adsorption-desorption isotherms of nitrogen at −196 °C and the pore size distribution for the powders calcined at 450 °C showed that the mesoporous structure with an average pore size of 2.5–6 nm with specific surface area (S_BET_) from 147 (pure TiO_2_) to 224 m^2^/g for manganese samples was formed. Increasing the concentration of dopant ions from 1 to 5% insignificantly influenced on S_BET_.

**Fig. 1:**
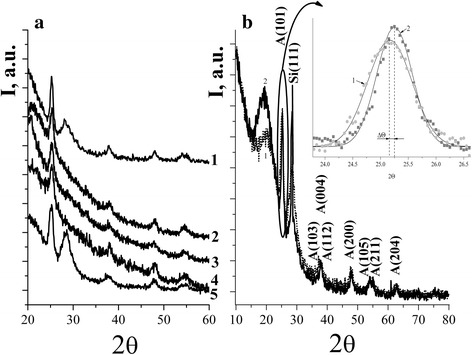
**a** XRD pattern of TiO_2_ (*1*), Co/TiO_2_ (*2*), Ni/TiO_2_ (*3*), Mn/TiO_2_ (*4*), and Cu/TiO_2_ (*5*) films doped with 5% of metal ions. **b** XRD patterns of TiO_2_ (*1*), Cu/TiO_2_ (5.5% Cu) (*2*) films and Gauss deconvolution of (101) peak (*inset*)

The diffraction peaks in the XRD patterns of films calcined at 450 °C (Fig. 1a) can be attributed to the anatase [ID = 71–1168]. The average size of crystallites was determined using Scherrer equation applied to the most intensive peak (101) of anatase using the Scherrer formula. For TiO_2_ film, it was 8 nm, while for Co/TiO_2_, Ni/TiO_2_, Mn/TiO_2_, and Cu/TiO_2_ (5% of dopant)—14, 14, 15, and 15 nm, respectively. Thus, the addition of transition metal ions in the precursor films of TiO_2_ accelerates crystallization and contributes to the growth of crystals, similar to results [15]. Similarity in the Ti^4+^, Ni^2+^, Co^2+^, Mn^3+^, and Cu^2+^ ionic radii (68, 69, 72, 80, and 72 pm, respectively) allows the interstitial incorporation of the dopant ions into the anatase lattice [15, 16]. In the case of copper-containing films, such inclusion leads to a shift in the peak of 101 in diffraction pattern (Fig. 1b).

**Fig. 2:**
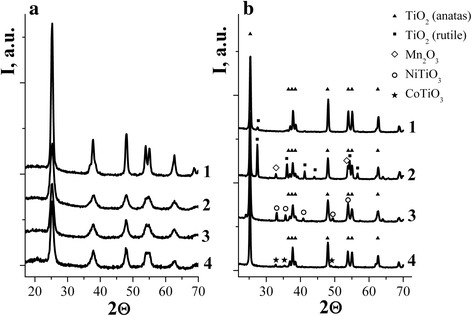
XRD patterns of Cu/TiO_2_ (*1*), Mn/TiO_2_ (*2*), Ni/TiO_2_ (*3*), and Co/TiO_2_ (*4*) powders doped with 5% of metal ions calcined at 450 (**a)** and 650 °C (**b)**

Figure 2a, b presented the structure of powders obtained from precursors of films that were calcined to 450 °C (a) and 650 °C (b). CoTiO_3_, NiTiO_3_, and Mn_2_O_3_ were detected in the XRD patterns of 5% Co/TiO_2_, Ni/TiO_2_, and Mn/TiO_2_ powders, respectively, when annealing temperature increased up to 650 °C. There were no displacements of the peaks of anatase or rutile in the XRD patterns of these powders, as it was observed for Cu/TiO_2_ films (≤5% Cu). Low-intensity phase reflexes of CuO, Cu_2_TiO_3_, and Cu_3_TiO_4_ for copper-containing systems were registered only at concentrations of Cu^2+^ ≥ 15%.

The presence of rutile phase was observed only in the samples of 5% Cu/TiO_2_ and 5% Mn/TiO_2_. The authors [17] have previously shown that the doping with manganese ions reduced the temperature of anatase-rutile phase transition. Concentration of rutile to the anatase in powder 5% Cu/TiO_2_ is negligible while in the manganese sample it was 41%.

### Optical Properties of Films and Powders M^n+^/TiO_2_

**Fig. 3:**
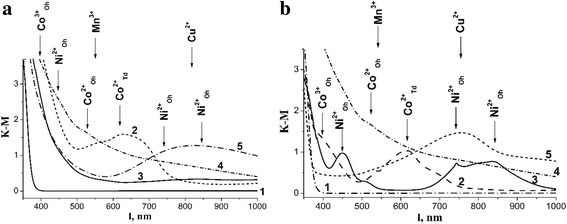
Diffuse reflectance spectra of the powders prepared from the films precursors: TiO_2_ (*1*) and TiO_2_ doped with 5% Co (*2*), Ni (*3*), Mn (*4*), and Cu (*5*) calcined at 450 (**a**) and 650 °C (**b**)

Obtained films were transparent, robust, and homogeneous. The refractive index and thickness of the films were measured by ellipsometry method. TiO_2_ film’s refractive index was 1.87 (2.55—for the bulk material), and the thickness was 64 nm. The smaller value of the refractive index of the films is due to the contribution of air (*n* = 1) to the developed pore structure in the effective refractive index of TiO_2—_air [18]. The refractive indexes of the films with Cu content of 5 and 20% are 1.94 and 1.66, and the thicknesses are 60 and 50 nm, respectively. The refractive indexes of the films containing 5% Co, 5% Mn, and 5% Ni were 1.92, 1.87, and 1.88, and the thicknesses were 95, 100, and 108 nm, respectively. Change of the thickness of the films can be attributed to a slight change in viscosity of the solution by adding an aqueous solution of salts of transition metals, as well as particularity of structure formation during annealing of the films. The absorption band in the UV region (λ = 380 nm) of diffuse reflectance spectra of the M^n+^/TiO_2_ powders after heat treatment at 450 °C (Fig. 3a) can be attributed to the band gap excitation of anatase TiO_2_ which corresponds to the band to band transition from Ti 3d to O 2p levels. [19]. There is a significant bathochromic shift of the absorption edge for all powders doped with transition metal ions. When transition metal ions incorporated into the lattice, the dopant level appeared between the valence band and the conduction band of TiO_2_, thus altering the band-gap energy and shift the absorbance edge to the visible light region [20]. There are the formation of additional energy levels in the band gap of TiO_2_ and decrease the E_g_ value induced by transition metal ions [20, 21, 22]. Such energy changes in a number of cases (at low concentrations of dopants) can increase the sensitivity of the photocatalyst in the visible region of the spectrum [19].

In the diffuse reflectance spectra of cobalt-containing powders of titanium dioxide absorption bands in the region of 650–800 nm (Co^2+^_Td_), 450–550 nm (Co^2+^_Oh_), and 350–440 nm (Co^3+^_Oh_), corresponding to d-d transitions in ions of cobalt of octahedral and tetrahedral coordination [21, 22] was observed (Fig. 3a, b curve 2). The absorption band at 350–440 nm is overlapped with the fundamental absorption band of titanium dioxide. According to [23], the presence of a band at 600–670 nm (Co^2+^_Td_), along with the characteristic absorption bands of Co^2+^_Oh_ and Co^3+^_Oh_, indicates the presence of spinel Co_3_O_4_ in samples. This d-transition is characterized by a high extinction which allows selecting a state of cobalt among others, even if it is a small amount [23].

According to [24], there are three allowed spin transitions from ^3^A_2g_ to ^3^T_2g_^3^T_1g_^3^T_1g_(P) that are located in the range of 770–1430, 500–910, and 370–525 nm for the systems of six-coordinated octahedral nickel (II). The state corresponding to forbidden spin transition to ^1^E_g_ lies close to the ^3^T_1g_, so their considerable mixing occurs. Therefore, the doublet band is observed in the spectrum. The optical spectra of Ni^2+^ is characterized by absorption at 410 and 730 nm, which correspond to nickel ions in octahedral environment (Ni^2+^_Oh_), and absorption at 525 and 650 nm, corresponding to Ni^2+^ ions in tetrahedral environment (Ni^2+^_Td_) [25]. The absorption band of Ni^2+^_Oh_, lying at 400–450 nm, is hard to distinguish from the fundamental absorption band of titanium dioxide (Fig. 3a, b curve 3). However, for the samples annealed at 650 °C, there is a distinct band with a maximum at 450 nm and a doublet at 750–850 nm. Three main characteristic absorption bands for nickel oxide (NiO) are observed at 407, 671, and 741 nm [24]. In the diffuse reflectance spectra of sample 5% Ni/TiO_2_ (650 °C), these bands are shifted to longer wavelengths (450, 743, and 837 nm, respectively), probably due to the distortion of the octahedral environment during the formation of nickel titanate (Fig. 3b).

The increase of Mn concentration in manganese-containing samples up to 5% (Fig. 3a, b curve 4) led to appearance of the shoulder at 550 nm in the diffuse reflectance spectra that corresponds to the transition in Mn^3+^ ions in an octahedral environment ^5^E_g_ → ^5^T_2g_ [19, 21]. Characteristic absorption bands of Mn^2+^_Oh_ and Mn^4+^_Oh_ [24, 26] in the short-wavelength region (450 nm) are overlapped with the fundamental absorption bands of titanium dioxide.

The broad structureless absorption band with a maximum at 800 nm was observed in the diffuse reflectance spectra of powders of Cu^2+^/TiO_2_. The absorption in the spectral region 600–1100 nm may be indicative for the existence of the copper (II) with a tetrahedral structure or a similar distorted structure (Fig. 3a, b curve 5) It is known that divalent copper ions have the electron configuration of 3d^9^. Ions with d^9^ configuration as a rule exhibit the stoichiometric mobility. Only one term ^2^D belongs to this configuration, which splits into two terms ^2^T_2_ and ^2^E in a cubic field. In a tetrahedral field, the lower level is the first term and in the octahedral—the second term. The observed peak can be attributed to the ^2^E_g_ → ^2^T_2g_ transition [19, 22, 27].

### Photoelectrochemical Characterization and Electrocatalytic Activity of M^n+^/TiO_2_ Films

**Fig. 4:**
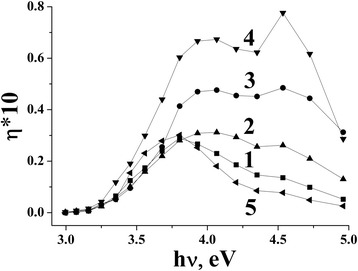
Quantum yield of photocurrent of 1% M^n+^/TiO_2_ electrodes: TiO_2_ (*1*), Co/TiO_2_ (*2*), Ni/TiO_2_ (*3*), Mn/TiO_2_ (*4*), and Cu/TiO_2_ (*5*)

Spectral dependences of photocurrent were measured for the TiO_2_ and M^n+^/TiO_2_ electrodes produced via coating of M^n+^/TiO_2_ films on Ti substrate (Fig. 4) to obtain the value of the band-gap energy. Photocurrent quantum yield for all of 1% M/TiO_2_ films is higher than that for undoped TiO_2_.

The enhancement of photocurrent efficiency indicated that M^n+^ ion addition is beneficial to promote charge separation within nanostructured TiO_2_ film and to improve interfacial charge transfer process due to formation of impurity electron levels of 3d metals in the band gap of titanium dioxide [21, 28, 29], acting as traps of charge which retard the recombination process. It is well known that quantum yield of photoelectrochemical current *η* in semiconductors can be expressed as [30]:

$$ \eta =\frac{A}{hv}{\left( hv-{E}_g\right)}^m $$

where *hν* is the photon energy, *m* = 1/2 for the direct transition, and *m* = 2 for the indirect transition. For the tested M^n+^/TiO_2_ compositions, photocurrent spectra were presented as (*η* × *hν)* ^*1/2*^ *= f(hν)* dependence which was linear in the wide range of wavelength. Experimental data fit better to an indirect transition. Band gap (E_g_) values were calculated [31, 32] by extrapolation of straight line of these dependences to the abscissa (Table 1). E_g_ values obtained by this method are located in the range of 2.88–3.09 eV (Table 1). For M/TiO_2_ films, the E_g_ values decrease with the increase of dopant content. That is probably associated with the formation of new phases. To test whether the narrower band gap is caused by a shift of the valence (E_vb_) or conduction band (E_cb_) edges, the position of the flat band potential (E_fb_) of the catalysts was determined by the direct electrochemical measurements of photocurrent as a function of applied potential in aqueous 0.5 NaCl. Flat band potentials were estimated from *i*_ph_ changes measured at the photocurrency maximum for TiO_2_ and M^n+^/TiO_2_ films coated on to titanium substrate in aqueous 0.5 M NaCl plotted against applied potential by extrapolation straight line of these dependences to the abscissa. Flat band potential values (Table 1) for the TiO_2_ differ insignificantly and are comparable with the value from −0.47 to −0.49 V vs NHE, obtained at pH ≈ 7 for nitrogen-doped titanium dioxide [33] and *E*_fb_ = −0.58 V measured for anatase single crystal [34].

**Table 1:** Photoelectrochemical characterization of M/TiO_2_ films (M–Co, Ni, Mn, Cu)

Sample	E_fb_ (eV) vs NHE	Quantum yield of photocurrent η (a.u.)	E_g_ (eV)	E_1/2_ (V)
TiO_2_	−0.42	1	3.09	−0.58
1% Ni/TiO_2_	−0.42	1.66	3.07	−0.46
5% Ni/TiO_2_	−0.45	1.14	2.95	−0.76
1% Co/TiO_2_	−0.36	1.07	3.07	−0.52
5% Co/TiO_2_	−0.80	0.28	2.97	−0.67
1% Mn/TiO_2_	−0.48	2.80	3.08	−0.52
5% Mn/TiO_2_	−0.40	0.93	2.88	−0.6
1% Cu/TiO_2_	−0.30	1.03	3.08	−0.55
5% Cu/TiO_2_	−0.15	1.14	3.07	−0.78
30% Cu/TiO_2_	−0.10	0.94	2.99	

As followed from Table 1, increase of M^n+^ content leads to the cathodic shift of the bottom of the conduction band Δ*E*_cb_ along with *E*_g_ decrease. The most significant changes of flat band potential values were observed for Cu-doped samples and 5% Co/TiO_2_ that probably related with coexistence of two valence states of dopant ions. As the location of the conduction band is a measure of the reduction power of the photogenerated electrons, we can predict the enhancing of catalytic activity in photoreduction processes.

**Fig. 5:**
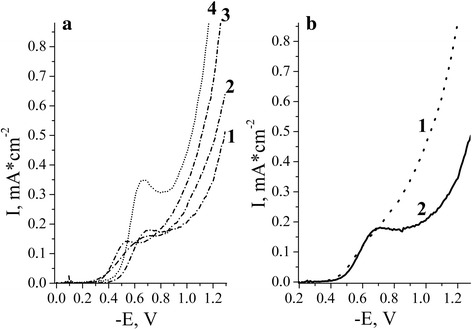
**a** Current-voltage curves for oxygen reduction in solution of 0.9% NaCl at the electrodes: (*1*)—TiO_2_, (*2*)—TiO_2_/Ni^2+^, (*3*)—TiO_2_/Co^2+^, (*4*)—TiO_2_/Cu^2+^ (1% of dopant). **b** Current-voltage curves for oxygen reduction in solution of 0.9% NaCl at the TiO_2_/Mn^3+^ electrode: (*1*)—first cycle, (*2*)—after cycling; *v =* 10 mV s^−1^

The oxygen electroreduction process was investigated in physiological (0.9%) solutions of NaCl. It has been found that oxygen reduction polarization curves of TiO_2_ electrodes doped with Co (1–5%), Ni (1–5%), Mn (1–5%), and Cu (1–5%) exhibited only one polarographic current wave at potentials of −0.40 to −0.9 V (against silver-chloride reference electrode) (Fig. 5). This suggests that the reaction proceeds without the formation of intermediate H_2_O_2_ [35, 36], the number of electrons involved in the electroreduction of oxygen on the M^n+^/TiO_2_ electrodes, is 2. For TiO_2_/Mn^3+^ (1%) sample, the polarization curves have not clearly defined threshold current (Fig. 5b, curve 1) but with the further potential cycling, their shape changes and takes the form characteristic of polarization curves for TiO_2_ with the limiting current −0.65–0.95 V (against silver-chloride reference electrode) (Fig. 5, curve 2).

The mechanism of oxygen reduction on the electrodes under investigation is determined by mixed kinetics. We have shown that the limiting oxygen reduction current for the semiconductor electrodes is diffusion current, the dependence of limiting current on dissolved oxygen concentration being linear.

The introduction of small amounts of a dopant (~1%) in the TiO_2_ film leads to an increase of catalytic activity, which manifests itself in the reduction half-wave reduction potential of O_2_ compared to unmodified samples. At a higher dopant content (>5%), the decreasing of catalytic activity for all 3d metals was observed, half-wave potential of oxygen reduction shifted in the cathodic region compared with TiO_2_ electrodes.

### Photocatalytic Activity of M^n+^/TiO_2_ Films

Photocatalytic activity of TiO_2_ and TiO_2_/Cu^2+^, TiO_2_/Co^2+^, TiO_2_/Mn^3+^, and TiO_2_/Ni^2+^ films has been tested in the photoreduction of toxic Cr(VI) to non-toxic Cr(III) ions in acid water solutions in the presence of environmentally important substrate EDTA as electron acceptor. This process has been taken as a model of real wastewaters, where oxidants and reductants are present together, for comparable studies of commercial samples and Pt/TiO_2_ powders [32]. The mechanism of photocatalytic Cr(VI) reduction in the presence of electron scavenger is well described in [37, 38]. Under irradiation in the presence of M^n+^/TiO_2_ films, the changes in Cr(VI) concentration was followed by decrease of absorption band intensity at 349 nm, simultaneously the absorption at 550 nm increases due to non-toxic Cr(III) formation. Dependence of Cr(VI) to Cr(III) first order rate constants on of the dopant concentration presented in Table 2.

**Table 2:** First order rate constants (*k′* × 10^5^, s^−1^) Cr(VI) to Cr(III) ions photoreduction in the presence of TiO_2_ and M^n+^/TiO_2_ (M–Co, Ni, Mn, Cu) films with different dopant concentrations

Sample	Concentration of dopant ions				
	0%	1%	3%	5%	7%
	First order rate constants (*k′* × 10^5^, s^−1^)				
Co/TiO_2_	3.3	4.4	4.1	4.4	3.8
Ni/TiO_2_		4.6	4.7	5.2	3.8
Mn/TiO_2_		4.5	4.2	3.5	3.6
Cu/TiO_2_		5.8	6.3	6.7	5.8

All doped films showed growth of photocatalytic activity compared with pure TiO_2_. The optimum dopant ion concentration is 1% for Co^2+^ and Mn^3+^; 5% for Cu^2+^and Ni^2+^. Creating the additional energy levels in the bandgap of semiconductor, dopant ions increase the time of recombination of photogenerated electrons and holes from the conduction and valence band of the TiO_2_ and increase the efficiency of charge separation.

## Conclusions

Nanostructured M^n+^/TiO_2_ films having mesoporous structure with an average pore size of 2.5–6 nm and specific surface area from 147 (pure TiO_2_) to 224 m^2^/g for manganese-containing samples were formed on glass and titanium substrates using template-assisted sol-gel method. After calcinations at 400 °C in the XRD patterns of annealed TiO_2_ films doped with nickel or cobalt ions, only anatase nanocrystalline phase (8–20 nm) was observed; incorporation of 5% manganese or cupper ions into TiO_2_ structure lead to the formation of rutile phase. The enhancement of photocurrent efficiency of metal-doped electrodes in comparison with undoped TiO_2_ indicates that M^n+^ ion addition is beneficial to promote charge separation within mesoporous TiO_2_ film and to improve interfacial charge transfer process. For TiO_2_ films doped with transition metal ions, the Eg values decrease with the increase of dopant content associated with the formation of new phases. Increase in M^n+^ content leads to the cathodic shift of the bottom of conduction band along with Eg decrease. M^n+^/TiO_2_ (1%M–Co, Ni, Mn, Cu) electrodes with low dopant content possess high efficiency in electrocatalytic reduction of dissolved oxygen. Polarization curves of TiO_2_, TiO_2_/Ni^2+^, TiO_2_/Co^2+/3+^, and TiO_2_/Mn^3+^ electrodes contain only one current wave current; it means that oxygen reduction proceeds without the formation of an intermediate product H_2_O_2_. The films containing 5 *w*/*w* % Cu, Mn, Ni, and Co exhibited the higher photoactivity in the processes of Cr(VI) to Cr(III) photoreduction comparing to TiO_2_ one. With increasing of dopant content, the decreasing of electrocatalytic activity and gradual decrease in the reaction rate constant of Cr(VI) to Cr(III) photoreduction for all 3d metals was observed. Synthesized covering can be used as effective photocatalysts and sensor elements.

## Abbreviations

E_cb_: Conductive band potential
E_fb_: Flat band potential
E_g_: Band gap
S_BET_: Specific surface area
UV light: Ultraviolet light
XRD: X-ray diffraction
